# Supplementary material for: Association between oral microbiome and seven types of cancers in East Asian population: a two-sample Mendelian randomization analysis
Source: Front Mol Biosci. 2023 Nov 21;10:1327893. doi: 10.3389/fmolb.2023.1327893 (PMC10702768; doi:10.3389/fmolb.2023.1327893)
Supplement: Supplementary file 1 [file DataSheet1.ZIP › Supplementary Materials/MR plots of saliva/saliva═╝/colorectal cancer/pheno.3648_to_colorectal cancer_leave_one_out.pdf]

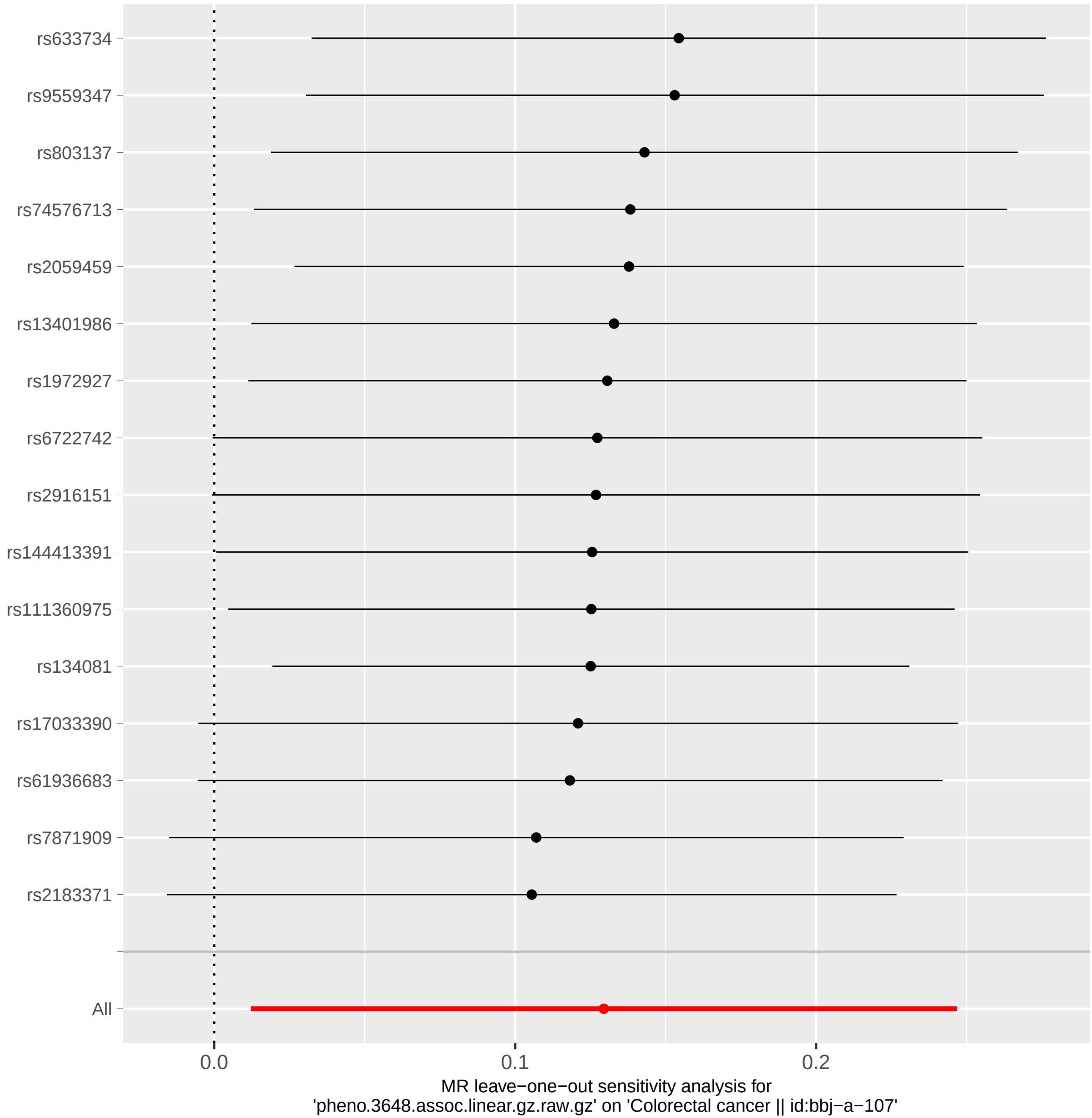
rs633734
rs9559347
rs803137
rs74576713
rs2059459
rs13401986
rs1972927
rs6722742
rs2916151
rs144413391
rs111360975
rs134081
rs17033390
rs61936683
rs7871909
rs2183371
All
0.0
0.1
0.2
MR leave−one−out sensitivity analysis for
'pheno.3648.assoc.linear.gz.raw.gz' on 'Colorectal cancer || id:bbj−a−107'
